# Patterns of postmastectomy radiation therapy in clinically node-positive breast cancer patients with pathologically negative lymph nodes after neoadjuvant chemotherapy

**DOI:** 10.3906/sag-2109-200

**Published:** 2021-11-13

**Authors:** Mutlay SAYAN, Irina VERGALASOVA, Mridula GEORGE, Maria KOWZUN, Lindsay POTDEVIN, Shicha KUMAR, Bruce HAFFTY, Nisha OHRI

**Affiliations:** 1 Department of Radiation Oncology, Dana-Farber Cancer Institute, Harvard University, Boston, MA, USA; 2 Department of Radiation Oncology, Rutgers Cancer Institute of New Jersey, New Brunswick, NJ, USA; 3 Department of Medical Oncology, Rutgers Cancer Institute of New Jersey, New Brunswick, NJ, USA; 4 Department of Surgery, Rutgers Cancer Institute of New Jersey, New Brunswick, NJ, USA

**Keywords:** Breast, carcinoma, radiotherapy, chemotherapy

## Abstract

**Background/aim:**

To analyze postmastectomy radiation therapy (PMRT) utilization and its association with overall survival (OS) in patients presenting with node-positive breast cancer who are pathologically node-negative (ypN0) after neoadjuvant chemotherapy (NAC).

**Materials and methods:**

Using the National Cancer Data Base (NCDB), we identified patients diagnosed between 2004 and 2013 with clinical T1–4 node-positive nonmetastatic breast cancer who received NAC and underwent mastectomy with pathologically negative lymph node sampling. Multivariable regression models identified factors associated with PMRT use. The Cox proportional hazards model was used to evaluate predictors of mortality.

**Results:**

The study included 8766 clinically node-positive patients who met the study criteria. PMRT was delivered to 61.5% of patients. Overall PMRT utilization rate increased over the study period from 54.4% in 2004 to 65.2% in 2011. Predictors of PMRT use included larger tumor size, increasing clinical N stage, higher grade disease, receipt of hormone therapy, and a greater number of lymph nodes examined. The unadjusted 5-year OS was 84.1% in the PMRT group and 83.8% in the non-PMRT group (p = NS). PMRT was not significantly associated with survival on multivariable analysis (hazard ratio [HR] 0.87; 95% confidence interval [CI] 0.73–1.03).

**Conclusion:**

The delivery of PMRT has increased over time in women presenting with clinically node-positive breast cancer who convert to ypN0 after NAC. While we identified multiple independent socioeconomic and clinical predictors of both PMRT utilization and survival, PMRT itself was not significantly associated with survival.

## 1. Introduction

The benefits of postmastectomy radiation therapy (PMRT) in locally advanced breast cancer after upfront surgical resection with or without adjuvant chemotherapy are well-established [1–3]. The role of PMRT in the setting of neoadjuvant chemotherapy, however, is an area of active investigation with limited available randomized data. There are two ongoing randomized clinical trials that will ultimately guide locoregional management [4]. The available data at this time suggests that both prechemotherapy clinical stage and postchemotherapy pathologic stage are predictive of locoregional recurrence [5–8]. Current National Comprehensive Cancer Network guidelines therefore recommend PMRT for patients with the residual nodal disease after NAC and state that PMRT should be “strongly considered” for patients with the upfront clinical nodal disease who are pathologically node-negative at the time of surgery (pN0).

With the increasing utilization of NAC among breast cancer patients, there is likely wide variation in PMRT practice patterns [9]. The current analysis sought to utilize the National Cancer Data Base (NCDB) to analyze PMRT practice patterns and survival outcomes among patients who are clinically node-positive at diagnosis and pathologically node-negative after NAC.

## 2. Materials and methods

### 2.1. Data source

The NCDB is a national hospital-based cancer registry that is a joint project of the Commission on Cancer (CoC) of the American College of Surgeons and the American Cancer Society (ACS). It is estimated that 70% of all newly diagnosed malignancies in the United States are captured by facilities participating in this registry and reported to the NCDB. Data are collected by participating cancer program’s registries and include details on patient characteristics, cancer staging, tumor histological characteristics, type of first-course treatment administered, and survival outcomes. The CoC’s NCDB and the hospitals participating in the NCDB are the sources of the deidentified data used in this study. The ACS and CoC have not verified and are not responsible for the analytic or statistical methodology employed or the conclusions drawn from these data. This study utilized deidentified data and was granted human research exemption from our institutional review board.

### 2.2. Patient selection

In this study, NCDB registry data from 2004 to 2013 were used to examine the delivery of PMRT to patients diagnosed with clinical T1–4 node-positive breast cancer (American Joint Committee on Cancer Staging Manual, sixth or seventh edition) who received NAC and underwent mastectomy with pathologically negative lymph nodes. Patients with clinical or pathologic evidence of distant metastatic disease, those with bilateral breast cancer, those who received intraoperative therapies, and those who did not receive any treatment at the reporting facility were excluded.

### 2.3. Treatments

NAC was defined by an interval from initiation of chemotherapy to surgery of 80 to 270 days. PMRT was defined as delivery of 45 Gray (Gy) or more of external beam radiotherapy to the chest wall with or without regional nodal irradiation. PMRT was required to start between 15 and 180 days after surgery. While the number of lymph nodes removed was reported in the NCDB, the axillary surgery type (sentinel lymph node biopsy vs. axillary dissection), the presence or absence of extranodal extension, and specific lymph node target volumes (axillary vs. supraclavicular vs. internal mammary lymph nodes) were not specified in the NCDB.

### 2.4. Statistical analysis

Chi-square tests were used to compare patient demographic, facility, clinicopathologic, and treatment details between patients who did and did not receive PMRT. Multivariate logistic regression was used to identify predictors of PMRT utilization. Five-year OS was estimated using the Kaplan-Meier method. Factors associated with mortality were identified using the Cox proportional hazards model. The patient, facility, and tumor level variables were considered in the analyses. A landmark of 180 days from surgery was used for survival analyses.

## 3. Results

**Figure 1:**
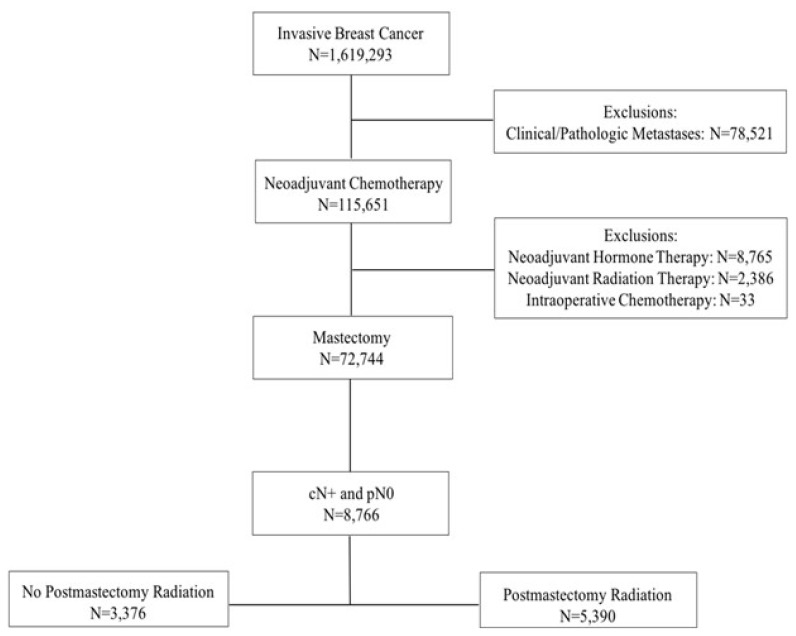
CONSORT diagram for 8766 patients with nonmetastatic invasive breast cancer who were clinically node-positive, received neoadjuvant chemotherapy, underwent mastectomy, and were pathologically node-negative.

A total of 8,766 breast cancer patients diagnosed between 2004 and 2013 with clinical T1–4 node-positive disease who received NAC and underwent mastectomy with pathologically negative lymph node sampling were identified. PMRT was received by 61.5% of these patients (Figure 1). Most patients (78.2%) had clinical N1 disease prior to NAC. Patients with clinical N2 and N3 were 13.4% and 8.5%, respectively. Prior to NAC, 53.4% of the patients had clinical T1–2 and 46.6% had clinical T3–4 disease. After NAC, 92.5% of the patients had pathologic T0–2 disease and 7.5% had pathologic T3–4 disease.

**Figure 2:**
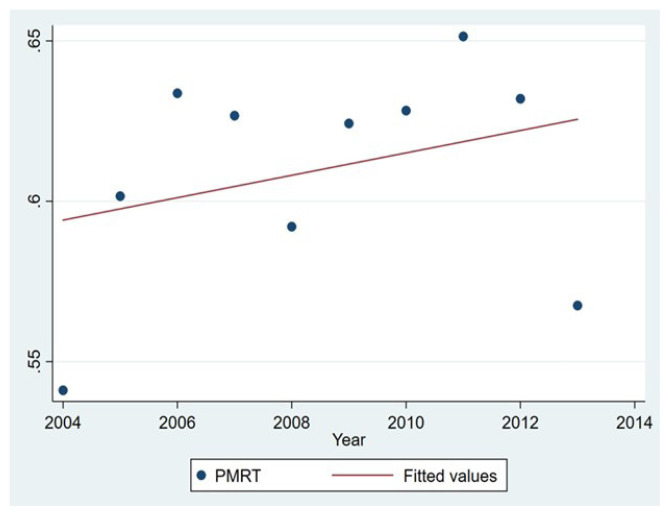
The use of postmastectomy radiation therapy is illustrated by the year of diagnosis.

Figure 2 demonstrates trends in PMRT use between 2004 and 2013. Overall PMRT utilization rate increased over the study period from 54.4% in 2004 to 65.2% in 2011. The rate of increase varied depending upon the year. The largest increase in PMRT utilization was seen between 2004 and 2005, from 54.4% to 60.2%.

Table 1 shows the predictors of PMRT utilization on multivariable analysis. Older age, higher Charlson-Deyo comorbidity score, insurance with Medicaid/Medicare, further distance from the treatment center, and reconstruction were all associated with decreased utilization of PMRT. Patients with higher clinical T stage, higher clinical N stage, or higher-grade disease were more likely to receive PMRT, as were patients who received hormone therapy and patients with greater numbers of lymph nodes examined. Race, income, facility type (academic vs. nonacademic), pathologic T stage, and laterality were not significantly associated with PMRT on multivariable analysis.

**Figure 3:**
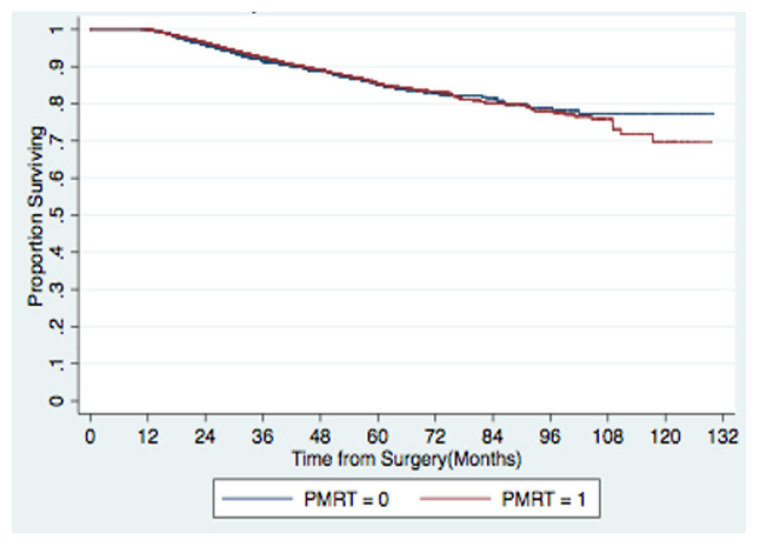
Kaplan-Meier 5-year survival curves for all patients stratified by PMRT utilization.

The Median follow-up was 39 months. Unadjusted 5-year overall survival was not significantly different at 84.1% in the PMRT group and 83.8% in the non-PMRT group (Figure 3). Table 2 lists the predictors of mortality according to multivariable analysis for all patients included in this study. Older age, increasing clinical or pathologic T stage, increasing clinical N stage, and Medicaid/Medicare insurance were associated with decreased survival. Patients who received hormone therapy or those with greater numbers of lymph nodes examined had improved survival. PMRT, race, Charlson-Deyo comorbidity score, income, facility type (academic vs. non-academic), laterality, and grade were not significantly associated with survival on multivariable analysis. Notably, PMRT was not significant associated with survival (multivariable hazard ratio [HR] 0.87; 95% confidence interval [CI] 0.73–1.03).

## 4. Discussion

With this study using a large population-based database, we have demonstrated that the utilization of PMRT increased in clinically node positive breast cancer patients who converted to pathologically node negative after NAC. PMRT utilization rates ranged from 54.4% to 65.2%, peaking in 2011. Though multiple independent predictors of PMRT utilization and survival were identified, PMRT itself was not significantly associated with survival.

Utilization of NAC has been increasing. Potential benefits include pathologic down-staging, avoiding delays in systemic therapy, and upfront treatment of micrometastatic disease [9–11]. These advantages, particularly in the setting of complete pathological nodal response, create a unique challenge for clinicians in determining the optimal subsequent locoregional therapy. While we await results of the ongoing NSABP B51/RTOG 1304 randomized clinical trial investigating the benefit of regional nodal irradiation in this patient population, the currently available data is largely limited by its retrospective nature and somewhat conflicting results.

One observation from our study is that PMRT was not significantly associated with survival. Similarly, Shim et al. analyzed the outcomes of 151 patients with clinical stage II (60%) and III (40%) breast cancer who were treated with NAC followed by mastectomy with complete pathological nodal response [12]. Of these, 105 received PMRT and 46 did not. The 5-year LRR-free survival was 98.1% with PMRT and 92.3% without PMRT, and the 5-year OS was similar at 93.3% vs. 89.9%. Le Scodan et al. also identified 134 women with clinical stage II (62%) and III (38%) breast cancer treated with NAC and mastectomy who were pathologically node-negative. PMRT was delivered to 78 patients. The 10-year LRR-free survival and OS rates were similar among patients who received PMRT and those who did not: 96.2% vs. 86.8% (p = 0.18) and 77.2% vs. 87.7% (p = 0.15), respectively [13]. Both studies reported that PMRT was not a prognostic factor on multivariate analysis. However, a study from MD Anderson Cancer Center showed that PMRT was associated with improved disease-specific and OS in breast cancer patients who achieved complete pathological response after NAC [14]. In this study, McGuire et al. identified 106 patients with clinical stage I (2%), II (31%), and III (66%) disease treated with NAC and mastectomy who had a complete pathological response. PMRT was delivered to 72 patients. In patients with clinical stage III disease, the 10-year LRR and OS rates were higher with PMRT compared to those without PMRT: 7.3% vs 33.3% (p = 0.040) and 77.3% vs 33.3% (p = 0.0016), respectively. However, the limited sample size and unbalanced baseline characteristics between the groups bring into question the statistical power of the reported study observations.

While the result of the ongoing NSABP B51/RTOG 1304 randomized clinical trial is pending, an unquantifiable factor that may have contributed to the increasing use of PMRT during the latter period of the study was the effect of the NCICMA.20 and EORTC 22922 trials. These trials examined the role of regional nodal irradiation in patients who had breast cancer with 1 to 3 positive lymph nodes. In 2010 and 2011, the preliminary findings from those trials suggesting that regional nodal irradiation improved disease-free survival and overall survival [15, 16]. While the majority of patients included in these trials underwent breast conserving therapy, these results are often extrapolated to patients who undergo mastectomy. The findings may have encouraged more radiation oncologists to pursue PMRT for women who had T1–T2 primary tumors with 1 to 3 positive lymph nodes. Furthermore, an important observation of our study was that the high rate of cN1 disease in the study population (78.2%) which may have contributed to no survival benefits with PMRT.

In the absence of randomized data, retrospective studies suggest that the initial extent of disease clinically and the response of axillary lymph nodes to NAC are important factors to consider when considering adjuvant PMRT. However, breast cancer patients who receive NAC represent a heterogeneous group, ranging from locally advanced, inoperable to early-stage, operable disease. Furthermore, different response rates to NAC also contribute to the heterogeneity of this patient population. Therefore, it is difficult to generalize treatment recommendations across such a diverse population. It is also not clear how to prioritize clinical stage at the time of presentation and residual pathologic disease burden after NAC in the decision-making process. It is likely this lack of clarity that contributes to the varying PMRT practice patterns seen in the present analysis.

There are several important limitations of our present analysis. Details regarding chemotherapy agents administered, the use of targeted therapies, the use of sentinel lymph node biopsy, radiation lymph node volumes, and biological characteristics of the tumor such as ER, PR, Her2, and Ki-67 status were not available. Additionally, the NCDB does not include data on LRR and disease-free survival, which is particularly relevant for our study population. Additional limitations of our study include the retrospective nature of the data, patient selection and institutional reporting bias, and a relatively short period of follow-up.

In conclusion, we report that about 60% of patients with clinically node-positive breast cancer who were ypN0 after NAC received PMRT. We identified multiple independent socioeconomic and clinical predictors of PMRT utilization. PMRT was not significantly associated with survival.

## Figures and Tables

**Table 1:** Predictors of postmastectomy radiation therapy receipt for all patients.

Variable*	Odds ratio	95% Cl	p
Age, y			
≤50	1.00	Reference	
>50	0.87	0.78–0.98	0.018
Charlson-Deyo score			
0–1	1.00	Reference	
2	0.64	0.42–0.99	0.046
Primary payer			
Private insurance	1.00	Reference	
Medicaid/Medicare	0.66	0.58–0.75	<0.001
Distance, miles			
>50	1.00	Reference	
≤50	0.70	0.58–0.85	<0.001
Hormone therapy			
No	1.00	Reference	
Yes	1.80	1.60–2.02	<0.001
Clinical T stage			
cT1–2	1.00	Reference	
cT3–4	1.83	1.64–2.06	<0.001
Clinical N stage			
cN1	1.00	Reference	
cN2	1.53	1.30–1.81	<0.001
cN3	2.02	1.63–2.49	<0.001
No. LNs examined			
<10	1.00	Reference	
≥10	1.22	1.09–1.36	<0.001
Grade			
G1	1.00	Reference	
G2	1.41	1.02–1.96	0.04
G3	1.47	1.02–2.02	0.02
Reconstruction			
No	1.00	Reference	
Yes	0.76	0.67–0.86	<0.001

* Only significant variables are shown. Race, income, facility type (academic vs nonacademic), pathologic T stage, and laterality were not significantly associated with PMRT utilization.

**Table 2:** Predictors of survival for all patients.

Variable*	Hazard ratio	95% Cl	p
Age, y			
≤50	1.00	Reference	
>50	1.34	1.12–1.61	0.002
Primary payer			
Private insurance	1.00	Reference	
Medicaid/Medicare	1.40	1.16–1.69	<0.001
Hormone therapy			
No	1.00	Reference	
Yes	0.56	0.50–0.69	<0.001
Clinical T stage			
cT1–2	1.00	Reference	
cT3–4	1.30	1.08–1.56	0.006
Clinical N stage			
cN1	1.00	Reference	
cN2	1.34	1.08–1.67	0.007
cN3	1.38	1.04–1.82	0.024
Pathologic T stage			
cT1–2	1.00	Reference	
cT3–4	1.61	1.27–2.03	<0.001
No. LNs examined			
<10	1.00	Reference	
≥10	0.70	0.59–0.83	<0.001
PMRT			
No	1.00	Reference	
Yes	0.87	0.73–1.03	0.115

* Only significant variables and PMRT are shown. Race, Charlson-Deyo score, income, facility type (academic vs. nonacademic), laterality, and grade were not significantly associated with survival.
